# Histone deacetylase 3 is required for development and metamorphosis in the red flour beetle, *Tribolium castaneum*

**DOI:** 10.1186/s12864-020-06840-3

**Published:** 2020-06-22

**Authors:** Smitha George, Subba Reddy Palli

**Affiliations:** Department of Entomology, University of Kentucky, Lexington, KY 40546 USA

**Keywords:** HDAC3, Juvenile hormone, *Tribolium castaneum*, Acetylation, Histone H3

## Abstract

**Background:**

Hormones are chemical communication signaling molecules released into the body fluids to stimulate target cells of multicellular organisms. We recently showed that histone deacetylase 1 (HDAC1) plays an important role in juvenile hormone (JH) suppression of metamorphosis in the red flour beetle, *Tribolium castaneum*. Here, we investigated the function of another class I HDAC member, HDAC3, and show that it is required for the normal development of *T. castaneum*.

**Results:**

RNA interference-mediated knockdown of the *HDAC3* gene affected development resulting in abnormally folded wings in pupae and adults. JH analog, hydroprene, suppressed the expression of *HDAC3* in *T. castaneum* larvae. The knockdown of *HDAC3* during the final instar larval stage resulted in an increase in the expression of genes coding for proteins involved in JH action. Sequencing of RNA isolated from larvae injected with dsRNA targeting *malE* (*E. coli* gene, control) or *HDAC3* followed by differential gene expression analysis identified 148 and 741 differentially expressed genes based on the *P*-value < 0.01 and four-fold difference, and the P-value < 0.05 and two-fold difference, respectively. Several genes, including those coding for myosin-I heavy chain (Myosin 22), Shaven, and nuclear receptor corepressor 1 were identified as differentially expressed genes in *HDAC3* knockdown larvae. An increase in histone H3 acetylation, specifically H3K9, H3K18, and H3K27, was detected in *HDAC3* knockdown insects.

**Conclusion:**

Overall, these data suggest that HDAC3 affects the acetylation levels of histones and influences the expression of genes coding for proteins involved in the regulation of growth, development, and metamorphosis.

## Background

Lysine acetylation is one of the major epigenetic modifications of proteins, which contributes to chromatin remodeling and expression of genes that regulate important biological processes [1]. In eukaryotes, the levels of acetylation of histones and other proteins are regulated by lysine acetyltransferases (KATs or Histone acetyltransferases HATs) and lysine deacetylases (KDACs or histone deacetylases HDACs), which catalyze the addition and removal of acetyl groups, respectively [2, 3]. Lysine acetylation targets large macromolecular complexes responsible for various nuclear and cytoplasmic cellular processes: such as splicing, cell cycle, chromatin remodeling, DNA replication, etc. [4]. HDAC enzymes depend on zinc ions for their catalytic activity, and human HDACs were grouped into four classes [5, 6]. Class I HDACs are localized in the nucleus, expressed universally, and play essential roles in cell proliferation, whereas class II and IV HDACs have a tissue-specific role [7, 8].

Recent studies using HDAC inhibitors have suggested multiple roles for HDACs in cell proliferation, cell cycle arrest, and apoptosis [9]. The knockdown of *HDAC3* induced changes in gene expression, DNA damage, and caused cell cycle delay in mouse embryonic fibroblasts [10]. In *Drosophila melanogaster*, six HDACs (Rpd3, HDAC3, HDAC4, HDAC6-S, HDAC6-L, and Sir2) were characterized by studying temporal expression patterns and transcriptional profiling and the effect of HDAC inhibitors [11]. The *D. melanogaster HDAC3* was cloned in 1998 and described as a metal-substituted enzyme [12]. RNA interference (RNAi)-mediated silencing of *HDAC1* or *HDAC3* in *Drosophila* S2 cells resulted in cell growth inhibition and deregulation of genes such as *sox14,* ecdysone-induced *eip74ef,* and *nvy* [13]. Chemical genomics studies revealed that HDAC1, 2 and 3 are essential for core regulatory transcription and cell proliferation in cancer models [14]. Deacetylation by HDAC3 plays a vital role in the suppression of apoptosis in *D. melanogaster* imaginal tissue [15]. Acetylation of specific lysine residues of histones contributes to the dynamic regulation of ecdysone induced genes in *D. melanogaster* [16]. However, the role of acetylation in the regulation of juvenile hormone (JH) action in insects is not well studied.

Juvenile hormones secreted by the corpora allata have multiple functions in an insect’s life cycle and regulate diverse biological processes, including larval development, molting, metabolism, polyphenism, diapause, reproduction, and metamorphosis [17–21]. The JH signals are transduced through JH receptor, Methoprene-tolerant (Met) [22, 23], Steroid receptor co-activator (SRC) [24], and CREB-binding protein (CBP) [25–27] (binding partners). JH represses the expression of genes involved in metamorphosis. *Kr-h1* is an early JH response gene downstream of *Met,* and RNAi mediated knockdown of Met or Kr-h1 induces a precocious larval-pupal transition in the red flour beetle [28]. JH/Met-dependent *Kr-h1* activity mediates the larval development. Lower JH titers result in lower levels of *Kr-h1* expression in the last instar larvae allowing expression of pupal specifier, Broad complex and adult specifier, E93 and metamorphosis [29].

Recent research from our lab showed that the class I and II HDAC inhibitor Trichostatin A (TSA) mimics JH in the induction of JH response genes [27], suggesting a role for HDACs in JH action. We also demonstrated that HDAC1 influences JH action by regulating acetylation levels of histones, which promotes the expression of JH response genes [30]. In the present study, we focused on another member of the class I HDAC family, HDAC3 (TC006104). Knockdown of the *HDAC3* gene during the final instar larval stage of the red flour beetle, *Tribolium castaneum* resulted in a pupa that showed abnormally folded wings and eventually died. RNA-seq analysis identified several genes including, Myo22, paired box protein Pax-5 (Shaven), and PDGF- and VEGF- related factor 3 (Pvf3), whose expression is influenced by HDAC3.

## Results

### HDAC3 plays a key role in development and metamorphosis

**Fig. 1:**
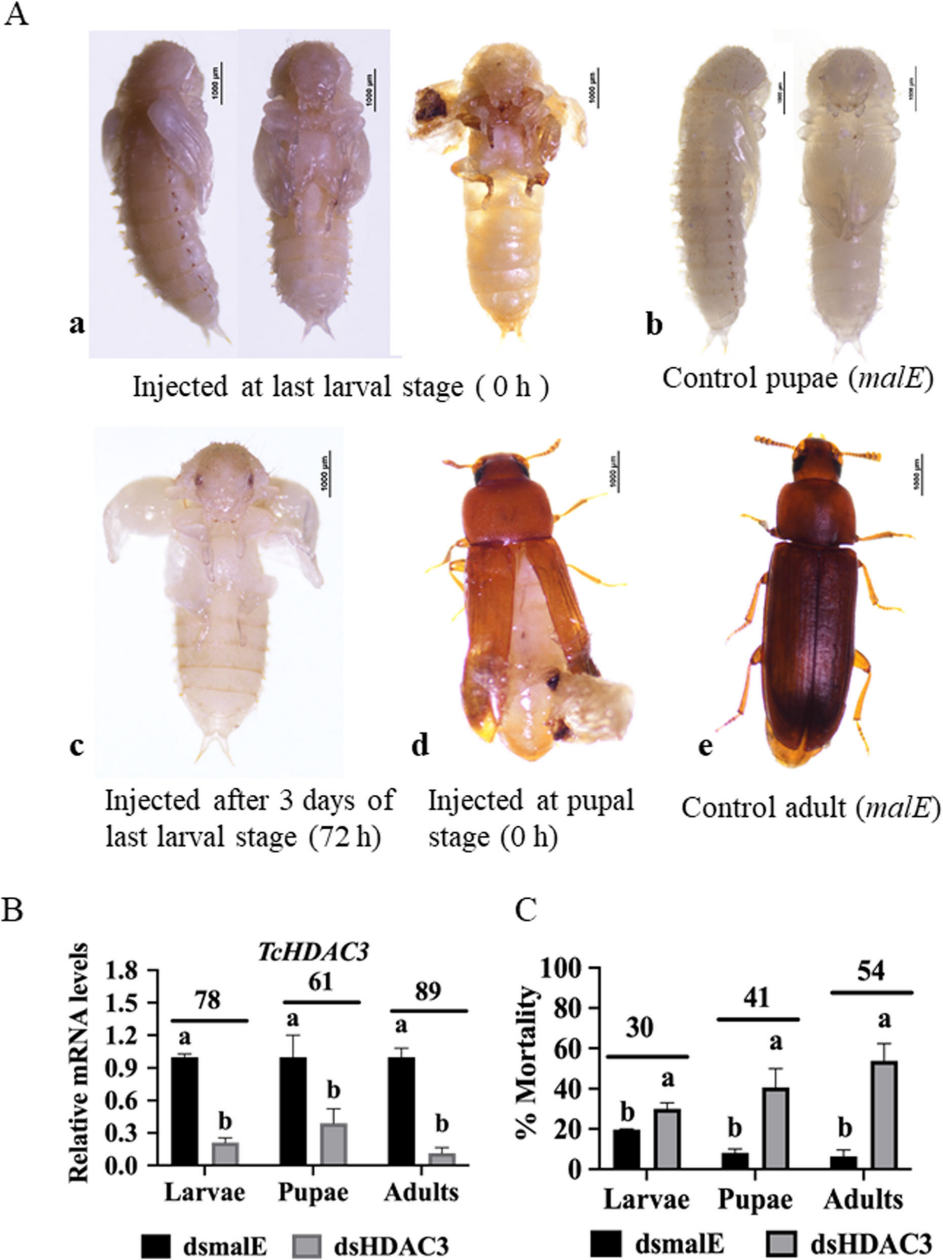
Phenotypes and mortality induced by RNAi-mediated knockdown of *HDAC3* in *T. castaneum*. A a) dsHDAC3 was injected into the newly molted last instar larvae. Developmental defects and mortality were recorded every day until adult eclosion. The knockdown of the *HDAC3* gene affected pupal development resulting in abnormally folded wings. b) Control larvae injected with dsmalE pupated in 5–6 days after injection and later emerged as healthy adults. c) The larvae injected with dsHDAC3 at 72 h after ecdysis to last instar larval stage pupated but showed abnormally folded wings. d) dsHDAC3 injected into newly formed pupae caused defects in the wing development. e) Healthy adults have emerged from the pupae injected with dsmalE. B HDAC3 mRNA levels were determined in larvae, pupae, and adults injected with dsHDAC3 or dsmalE. dsRNA were injected into day 0 last instar larvae, pupae and adults and the insects were collected on the third day after treatment, total RNA extracted and used to determine relative HDAC3 mRNA levels. Levels not connected by the same letter are significantly different. Mean ± SE (*n* = 30) are shown. C Injection of dsHDAC3 into day 0 last instar larvae, pupae and adults induced 30, 41 and 54% mortality, respectively mortality not connected by the same letter are significantly different. Mean ± SE (n = 30) are shown

HDAC3 is a member of the Arginase/deacetylase superfamily that belongs to class I and is structurally and functionally related to HDAC1 and HDAC8 (Additional file 1, Fig. S1. A). Orthologues of HDAC3 are present in insects, other arthropods, and vertebrates (Additional file 1, Fig. S1. B, Gregoretti, Lee, and Goodson 2004). Injection of one microgram of dsRNA into newly molted last instar larvae induced 30% larval mortality by eight days after dsRNA injection. The remaining larvae pupated but showed wing abnormalities, especially with wing folding, and could not complete development to the adult stage (Fig. 1Aa). Control larvae injected with dsmalE (dsRNA targeting *malE* gene from *Escherichia coli*) developed into normal pupae (Fig. 1Ab). Similarly, pupae with wing defects were observed when dsHDAC3 was injected into 72 h-old (day 3) last instar larvae (Fig. 1Ac). Also, adults developed from pupae injected with dsHDAC3 showed wing defects (Fig. 1Ad). The pupae that developed from dsHDAC3 treated larvae are smaller in size than the control larvae treated with dsmalE (Additional file 1, Fig. S2). Conversely, dsmalE injected pupae developed into normal adults (Fig. 1Ae). Injection of dsHDAC3 into larvae, pupae and adults induced 78, 61 and 89% of knockdown of target gene respectively in larvae, pupae and adults (Fig. 1B) and resulted in 30, 41 and 54% mortality, respectively in larvae, pupae and adults (Fig. 1C).

### Expression of *HDAC3* in larval and pupal stages

**Fig. 2:**
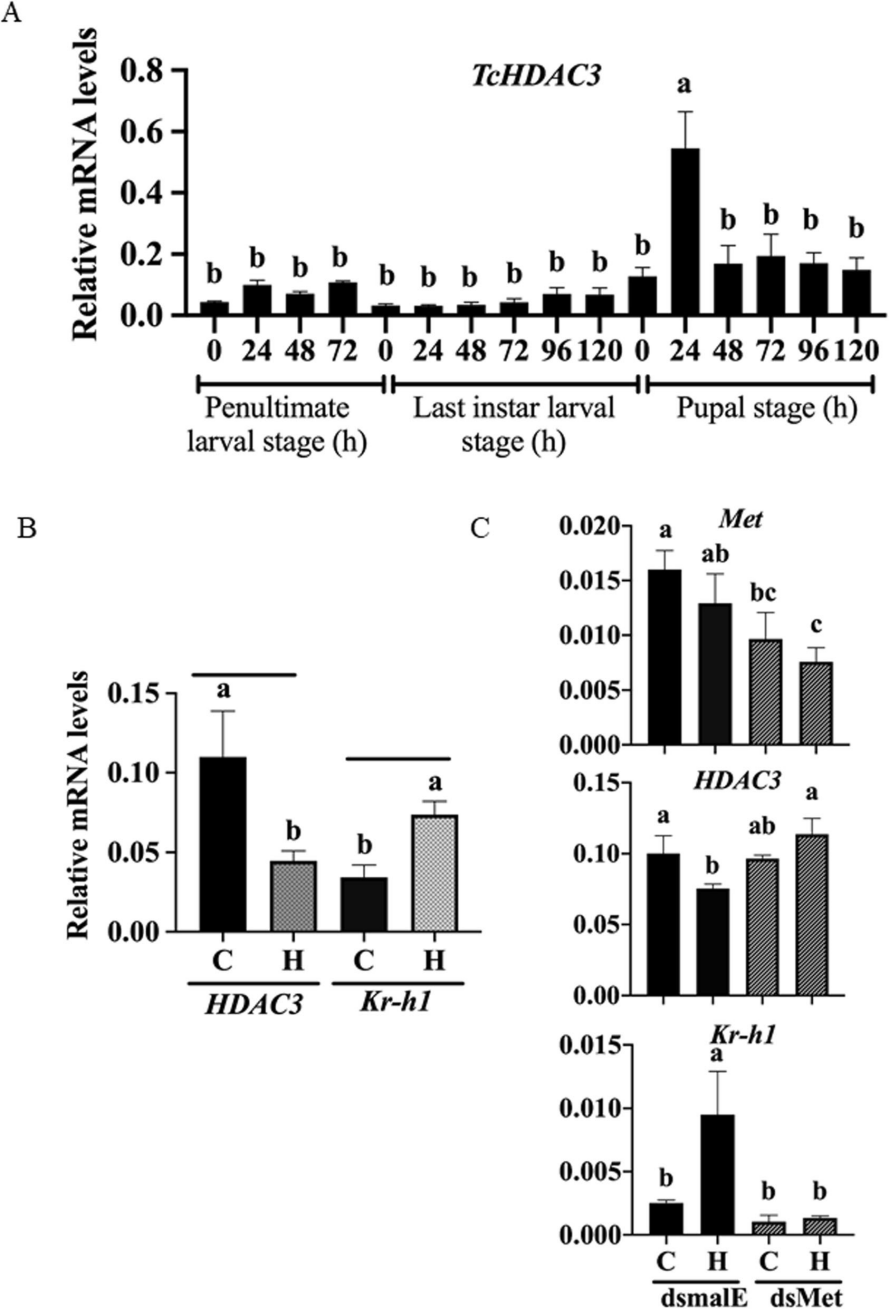
Developmental expression and JH induction of *HDAC3* in *T. castaneum. A HDAC3* mRNA levels were determined during the penultimate, last larval, and pupal stages at 24 h intervals. Total RNA isolated from a pool of two larvae for each replication was converted to cDNA and used in RT-qPCR to determine the relative HDAC3 mRNA levels. Mean ± SE (*n* = 4) are shown. Levels not connected by the same letter are significantly different. B JH suppresses the expression of *HDAC3* in *T. castaneum* larvae. S-Hydroprene (H, JH analog) was dissolved in cyclohexane (C) and topically applied to 48 h-old last instar larvae (0.5 μL of 2 μg/μL). At six hours after treatment, total RNA was isolated and subjected to RT- qPCR. The expression of the JH response gene *Kr-h1* was significantly induced, and *HDAC3* was significantly suppressed, mean ± SE (n = 4) are shown. Levels not connected by the same letter are significantly different. C Met is required for suppression of *HDAC3* by hydroprene. Newly molted last instar larvae were injected with dsMet or dsmalE. At 48 h after injection of dsRNA, the larvae were treated with hydroprene. Total RNA isolated from larvae was converted to cDNA and used to quantify *Kr-h1, HDAC3* and, *Met* mRNA levels. The data shown are mean ± SE (n = 4). The data were analyzed using analysis of variance, each pair student’s *t*-test. Mean values with the same letter are not significantly different from each other. C, cyclohexane; H, hydroprene

Developmental expression of *HDAC3* during the penultimate and last instar larval and pupal stages was determined using reverse transcription-quantitative PCR (RT-qPCR) and *HDAC3*-specific primers (Additional file 1, Table S1). The HDAC3 mRNA levels were low during the penultimate and last instar larval and early pupal stages but increased at 24 h after pupal ecdysis. (Fig. 2A). The HDAC3 mRNA levels then decreased again, and lower levels were maintained throughout the pupal stage. In general, the HDAC3 mRNA levels were higher during the pupal stage when compared to those during the penultimate and last instar larval stages.

### JH analog hydroprene suppresses the expression of *HDAC3* in *T. castaneum* larvae

The HDAC3 mRNA levels were significantly lower in hydroprene treated larvae when compared to those in control larvae treated with solvent (Fig. 2B). As expected, the mRNA levels of JH response gene *Kr-h1* increased in hydroprene treated larvae when compared to those in control larvae treated with cyclohexane (Fig. 2B). Also, the difference in expression levels of HDAC3 in larvae, pupae and adults was detected (Additional file 1, Fig. S3). Higher HDAC3 mRNA levels were detected in wing discs when compared to the other tissues isolated from 72 –h-old last instar larvae (Additional file 1, Fig. S3). In contrast, no significant differences in HDAC3 mRNA levels were detected in different tissues dissected from pupae (Additional file 1, Fig. S3).

To determine whether the JH receptor, Methoprene tolerant, Met, mediates JH suppression of *HDAC3*, we injected dsMet into last instar larvae and treated them with hydroprene or cyclohexane. As expected, the HDAC3 mRNA levels decreased in dsmalE (control) injected larvae treated with hydroprene but not in dsMet injected larvae treated with hydroprene (Fig. 2C). Also, *Kr-h1* mRNA levels increased in dsmalE (control) injected larvae treated with hydroprene but not in dsMet injected larvae treated with hydroprene (Fig. 2C). These data suggest that Met is required for JH III suppression of *HDAC3* gene expression.

### Knockdown of *HDAC3* induces expression of genes involved in JH action and response in *T. castaneum* larvae and pupae

**Fig. 3:**
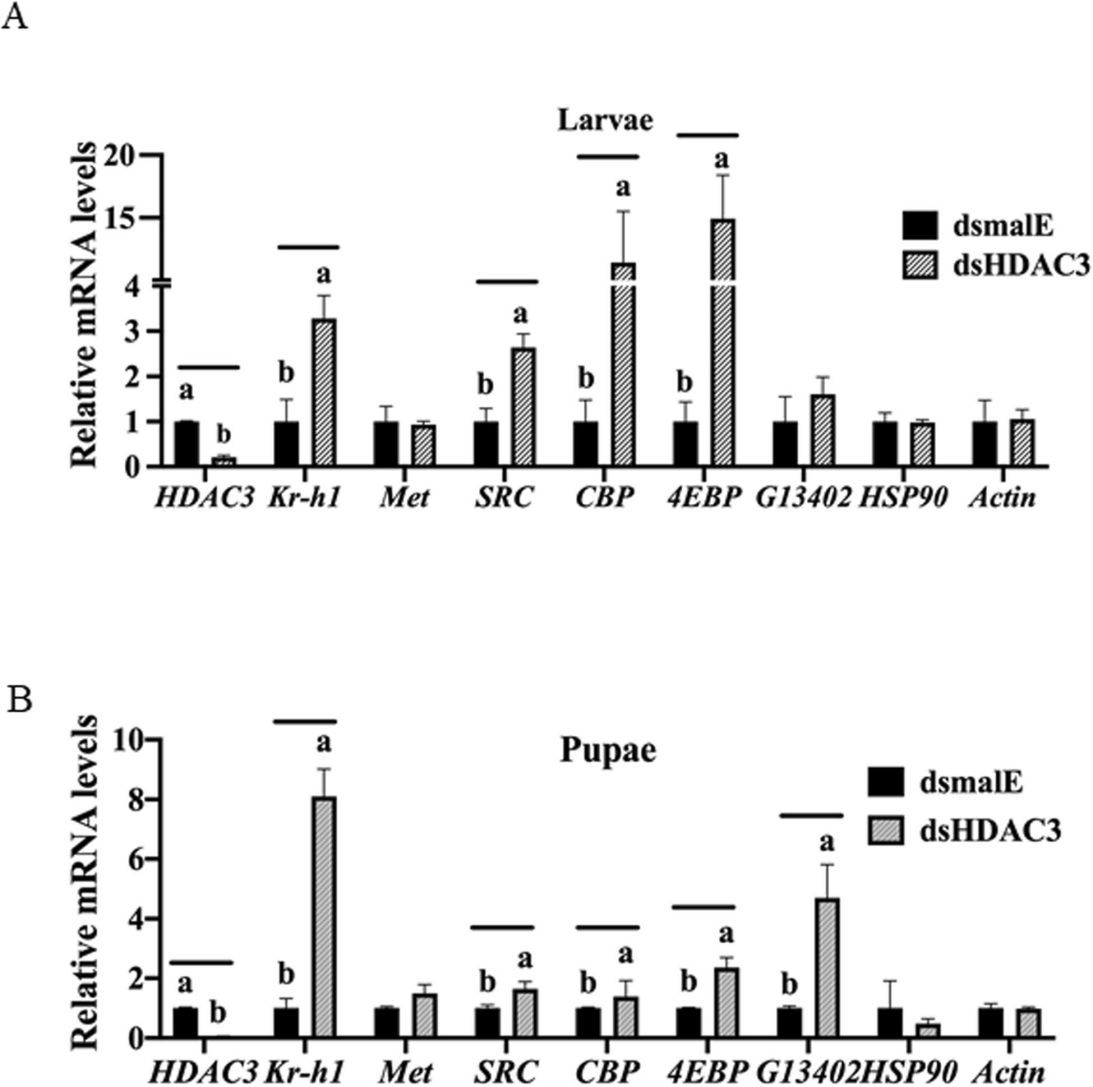
*HDAC3* knockdown in the last instar larvae of *T. castaneum* affects the expression of genes involved in JH action and response. A The knockdown of *HDAC3* in newly molted last instar larvae caused an upregulation of genes involved in JH action (*SRC, CBP*) and JH response (*Kr-h1, 4EBP, G13402*). Newly molted last instar larvae were injected with dsHDAC3 or dsmalE. Total RNA was extracted at 12 h after treatment, and the mRNA levels of JH-response genes (*Kr-h1, 4EBP)* genes involved in JH action (*Met, SRC, CBP*), HSP90 and Actin were quantified. The mean ± SE (n = 4) are shown. The data were analyzed using analysis of variance, each pair student’s *t*-test. Mean values with the same letter are not significantly different from each other. B The knockdown of *HDAC3* in pupae caused an upregulation of JH response genes (*Kr-h1, 4EBP, G13402*). 72 h-old last instar larvae were injected with dsHDAC3 or dsmalE. Total RNA was extracted on the fifth day after injection was used to determine relative mRNA levels of *SRC, CBP*, *Kr-h1, 4EBP, G13402, HSP90,* and *Actin*

*HDAC3* knockdown efficiency and its effect on the expression of JH response genes were tested using RT-qPCR. A significant knockdown of *HDAC3* was detected in larvae collected at 12 h after dsHDAC3 injection (Fig. 3A). The Kr-h1, 4EBP, SRC, and CBP mRNA levels increased significantly in dsHDAC3 injected larvae when compared to those in dsmalE injected larvae. The expression of *Met* was not affected by *HDAC3* knockdown. We also tested the housekeeping genes actin and heat shock protein (*HSP90*) to determine whether this effect is universal. Actin and *HSP90* mRNA levels were not affected by *HDAC3* knockdown (Fig. 3A). A similar pattern of *HDAC3* knockdown and an increase in the expression of *Kr-h1, 4EBP, and SRC* were detected in 24 h-old pupae developed from dsHDAC3 injected larvae (Fig. 3B). The CBP mRNA levels did not increase in pupae developed from dsHDAC3 injected larvae. Also, the mRNA levels of the JH-response gene, G13402 did not increase in dsHDAC3 injected larvae (Fig. 3A) but increased in pupae developed from dsHDAC3 injected larvae (Fig. 3B).

**Fig. 4:**
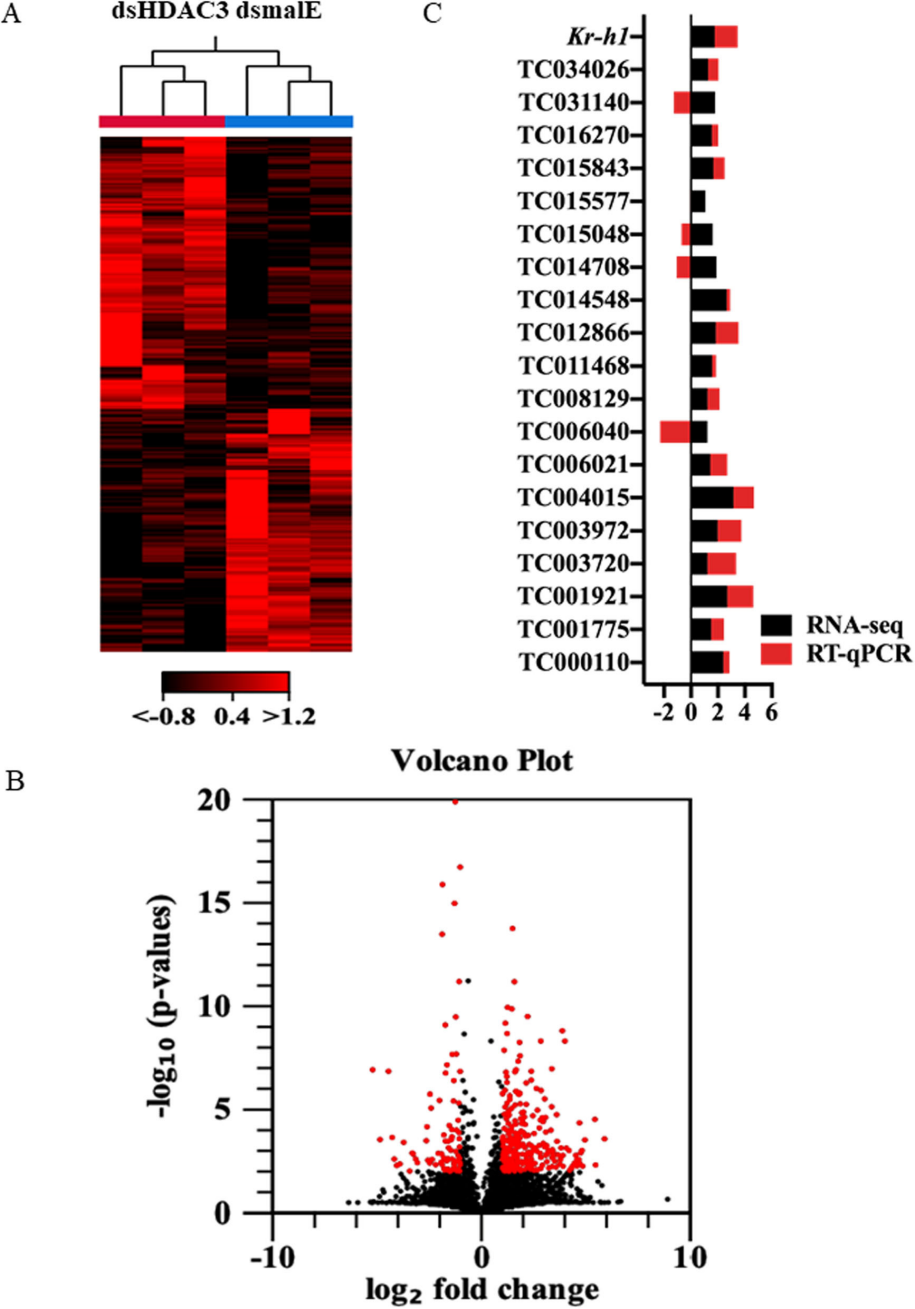
*HDAC3* knockdown in the last instar larvae of *T. castaneum* affects the transcription of genes involved in multiple pathways. A Heatmap of RNA-seq data. The heatmap of normalized mean expression values of 741 differentially expressed genes (≥ 2- fold and a *P* < 0.05) between dsmalE and dsHDAC3 treated insects. B Differentially expressed genes identified after *HDAC3* knockdown represented as the volcano plot. The X and Y-axis represent the -log_10_*P*- values and log_2_ fold change of mean normalized values, respectively. The red dots indicate the genes that showed ≥2- fold difference in expression with a *P* < 0.05. C RT-qPCR verified the expression of 20 selected genes from the up-regulated group (RNA-seq data). Descriptions of genes are listed in (Additional file 1, Table S4)

To identify other target genes whose expression is affected by *HDAC3* knockdown, we sequenced the RNA isolated from dsHDAC3 and dsmalE injected larvae. Run summary and read count statistics of sequencing output are shown in Additional file 1, Table S2. The overall pattern of normalized mean expression values of differentially expressed genes (DEGs) is represented as a heatmap (Fig. 4A). The DEGs are shown as a volcano plot with red dots indicating statistically significant genes after the EDGE test between treatment and control (Fig. 4B). After statistical analysis using Baggerley’s test to compare gene expression between dsHDAC3 and dsmalE treated insects, we identified 148 and 741 DGEs based on the *P*-value < 0.01 and four-fold difference, and the *P*-value < 0.05 and two-fold difference, respectively (Additional files 2 & 3). Among these, 126 and 563 genes were up-regulated, and the rest of them were down-regulated under the two stringency conditions tested. Hormone response genes, *Kr-h1*, Ecdysone induced protein 78C, and broad complex were up-regulated in *HDAC3* knockdown larvae (Additional file 1, Table S3). Web-based GO analysis of differently expressed genes showed enrichment of GO terms for binding, especially nucleic acid and ion binding, regulation of the cellular process, biological regulation, and transport (Additional file 1, Fig. S4).

Twenty genes (Additional file 1, Table S4) that are up-regulated in both *HDAC3* and *HDAC1* knockdown larvae [30] were selected for verification of RNA-seq data DEG predictions by RT-qPCR. The genes were selected based on the presence of a DNA-binding domain with possible functions as transcription factors, and RT-qPCR was used to determine their mRNA levels. Sixteen out of 20 genes tested showed an increase in their mRNA levels in *HDAC3* knockdown larvae when compared to those in control dsmalE treated larvae (Fig. 4C). Comparison of up-regulated genes between JH III [31] and dsHDAC3 treated larvae identified six common genes, including *Kr-h1* (Additional file 1, Table S5). Six genes that code for proteins containing zinc finger COG5048 domains found in *Kr-h1* were also up-regulated in *HDAC3* knockdown larvae (Additional file 1, Table S6).

### Identification of genes affected by both *HDAC3* knockdown and TSA treatment

**Fig. 5:**
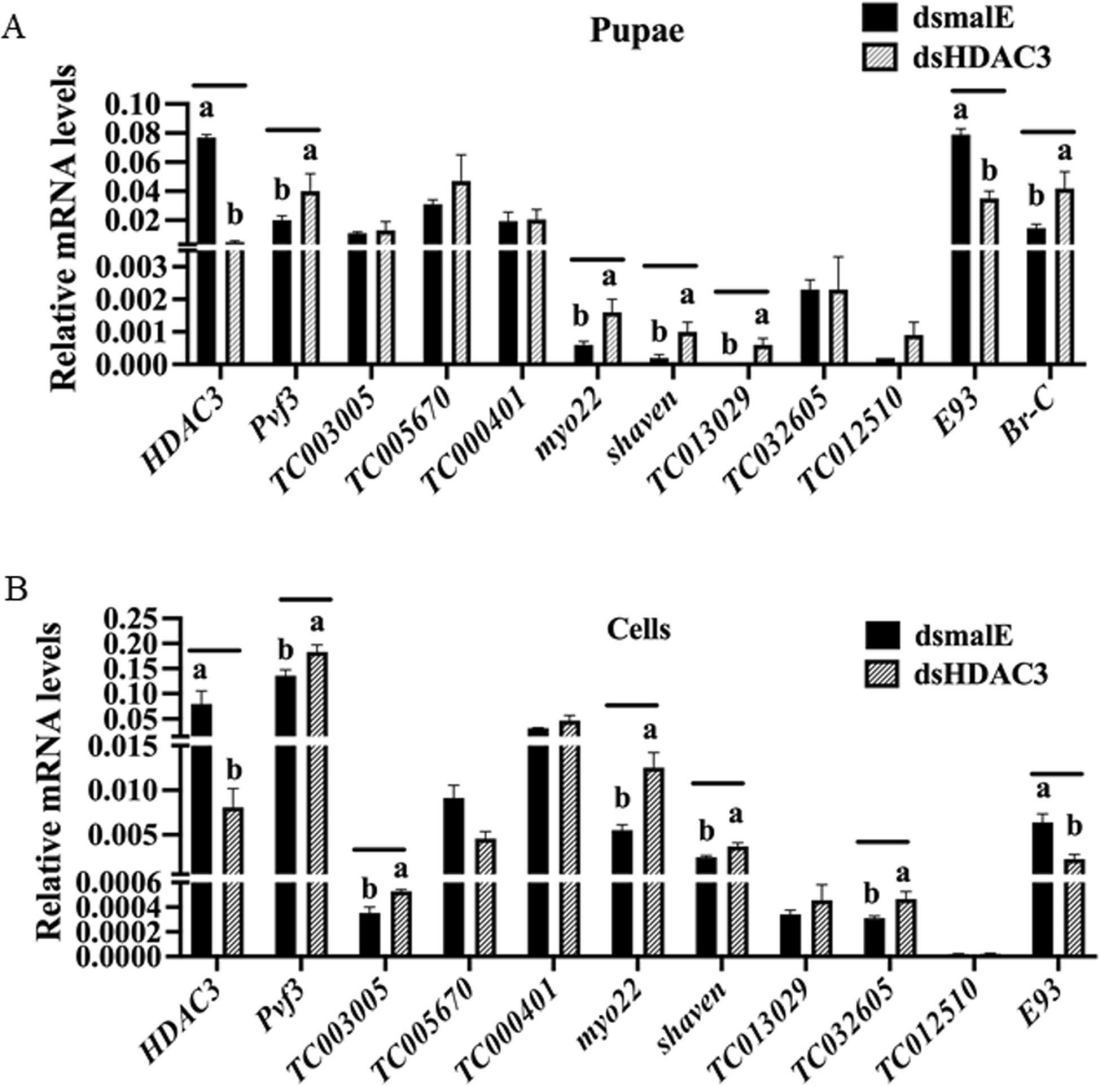
Confirmation of *HDAC3* targets in *T. castaneum* pupae and TcA cells. A 72 h-old last instar larvae were injected with dsHDAC3 or dsmalE. Total RNA was extracted at five days after injection, and mRNA levels were quantified. The mean ± SE of four replications is shown. The data were analyzed using analysis of variance, each pair student’s *t*-test. Mean values with the same letter are not significantly different from each other. TcA cells were treated with dsHDAC3 or dsmalE. Total RNA was extracted 72 h after dsRNA treatment, and mRNA levels were quantified. The mean ± SE of four replications is shown. The data were analyzed using analysis of variance, each pair student’s *t*-test. Mean values with the same letter are not significantly different from each other

TSA selectively inhibits class I and II HDACs and was shown to alter gene expression by preventing the removal of acetyl groups from histones [32]. Previous studies from our lab identified TSA induced genes in *T. castaneum* TcA cells [31]. Comparison of TSA induced genes with up-regulated genes in *HDAC3* knockdown insects identified multiple genes (5.3% of DGEs) that are common in both the treatments (Additional file 1, Fig. S5). The common genes identified from this analysis are listed in Additional file 4. To verify the results, we selected nine genes from this list (Additional file 1, Table S7) and determined their mRNA levels in dsHDAC3 treated *T. castaneum* pupae (Fig. 5A) and TcA cells (Fig. 5B). Myo22 (myosin-I heavy chain/TC008923), shaven (paired box protein Pax-5/TC003570) and Pvf3 (PDGF- and VEGF-related factor 3/TC008417) were significantly up-regulated in dsHDAC3 treated *T. castaneum* pupae and TcA cells when compared to their expression in control insects and cells treated with dsmalE (Fig. 5A, B). We also confirmed the significantly higher levels of neprilysin-11 (TC013029) in pupae treated with dsHDAC3 when compared to that in control pupae treated with dsmalE (Fig. 5A). Also, zinc finger protein 2-like (TC032605) and muscle M-line assembly protein unc-89 (TC003005) were significantly up-regulated in TcA cells treated with dsHDAC3 (Fig. 5B). Since HDAC3 deacetylates co-activators like acetyltransferases p300/CBP, p300/CBP-associated factor (PCAF) [33, 34], we compared lists of TSA induced genes, up-regulated genes in *HDAC3* knockdown insects and down-regulated genes from CBP knockdown cells [26]. Common genes identified from this comparison are listed in Additional file 1, Table S8.

### HDAC3 regulates acetylation levels of histone H3

**Fig. 6:**
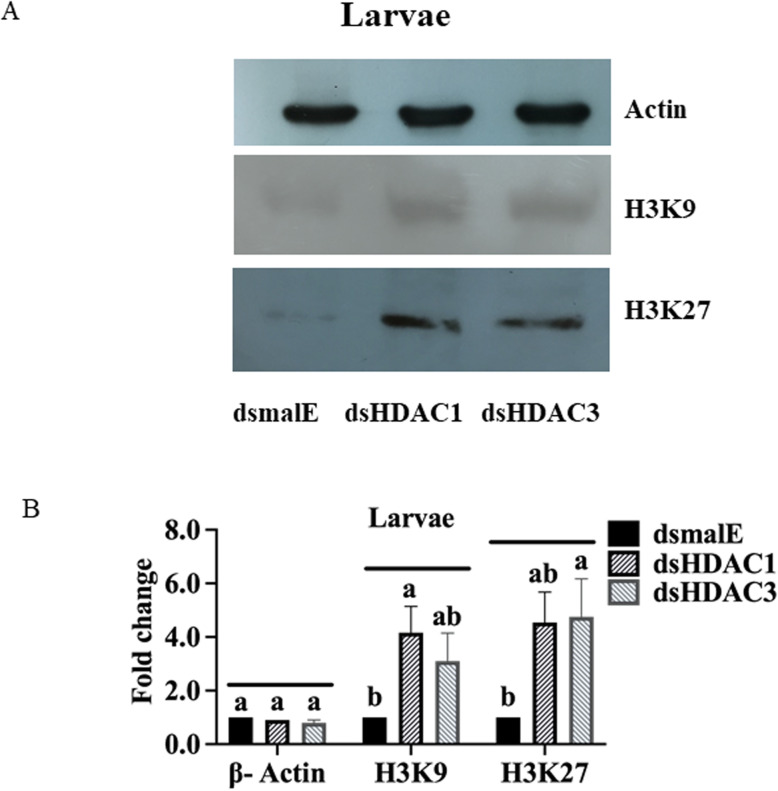
*HDAC3* knockdown affects acetylation levels of histone H3 in *T. castaneum* larvae. A The knockdown of *HDAC1* and *HDAC3* increased acetylation levels of histone H3. Total protein extracted from dsHDAC1, dsHDAC3 or dsmalE injected larvae were separated on SDS-PAGE gels, transferred to western blots, and hybridized with antibodies recognizing Acetyl-Histone H3 (Antibody Sampler Kit # 9927-Cell Signaling. ß-actin served as a loading control. The HRP-linked IgG (#7074-Cell Signaling) was used as a secondary antibody. The lysine acetylation status of histone H3K9 and H3K27 increased in *HDAC3* knockdown larvae. The images of complete Western blots are shown in Additional file 5. B Loading control protein, ß-Actin, was used to normalize the levels of H3K9 and H3K27. Band intensities were determined by Image-J software. The fold change in treatments compared to control was represented in the graph

Total proteins were isolated from the dsHDAC3-treated last instar larval tissues and subjected to the western blot assay using acetyl-histone H3 antibody sampler kit #9927 (Cell Signaling, MA) to determine the targets of *HDAC3* deacetylation. We evaluated the various lysine acetylation sites of histone H3 using Lys9, Lys14, Lys18, Lys27, and Lys56 specific antibodies. Increased acetylation of H3K9 and H3K27 was detected in dsHDAC1, and dsHDAC3 treated larvae compared to their levels in dsmalE treated larvae (Fig. 6A, B). These data suggest that H3 is one of the targets for HDAC1 and HDAC3.

## Discussion

Recent research in our laboratory demonstrated that HDAC1 suppresses *Kr-h1* gene expression and regulate JH suppression of metamorphosis in *T. castaneum* [30]. In the current studies, we investigated the role of the other member of the HDAC class I, the HDAC3. Unlike HDAC1 knockdown, which causes complete lethality during the larval stage, some of the *HDAC3* knockdown larvae undergo pupation, but the pupae exhibited defects, especially wing folding and the pupae that developed from dsHDAC3 treated larvae are smaller in size compared to the control larvae treated with dsmalE (Additional file 1, Fig. S2). Injection of dsHDAC1 into *T, castaneum* induced a block in growth and development and 100% mortality of larvae before pupation [30].

In contrast, HDAC3 knockdown is less severe, and some of the treated larvae completed larval development and died during the pupal stage. Some of the differences may be due to differences in the expression pattern of these two HDACs during the last larval stage. Further research is needed to uncover differences in the function of these two Class 1 HDACs. In *D. melanogaster*, mutations in *HDAC3* caused death during the late third instar larval and early pupal stages. Also, the imaginal discs are significantly reduced, and the pouch region of the wing disc was smaller in size compared to the wild-type [15]. RNAi-mediated *HDAC3* knockdown in the beetle, *Gnatocerus cornutus*, caused a reduction in hind wing size [35].

One of the primary outcomes of this research is the discovery that HDAC3 is required for normal larval, pupal and adult development in *T. castaneum*. The knockdown of *HDAC3* in newly molted last instar larvae caused an upregulation of genes involved in JH action (*SRC, CBP*) and JH response (*Kr-h1 and 4EBP*). In *D. melanogaster,* HDAC3 plays a crucial role in development, consistent with their relatively high expression during the embryonic and adult stages [11]. Our developmental expression studies showed a significant upregulation of the *HDAC3* gene expression in 24 h-old pupae (Fig. 2A). Previous studies reported that *T. castaneum HDAC3* is expressed during all developmental stages, and the highest mRNA levels were detected in one-day-old pupae [36]. We also demonstrated that the depletion of HDAC3 during the pre-pupal stage (72-h old) *T. castaneum* results in abnormal pupal development. Interestingly, transcription factor E93 (adult specifier) was down-regulated in dsHDAC3 knockdown pupal and cell samples (Fig. 5A, B). In *T. castaneum* and *D. melanogaster,* E93 promotes adult metamorphosis and represses the expression of antimetamorphic genes *Kr-h1* and pupal specifier, Broad*-complex* [37]. In *HDAC3* knockdown insects, we found up-regulation of *Kr-h1, Br-C,* and downregulation of *E93*. The timing and expression levels of *Kr-h1, Br-C,* and *E93* (metamorphic genetic network) facilitate the proper larval-pupal-adult transition in holometabolous insects [38]. Based on these data, we propose that the misregulation of critical hormone-related genes caused the abnormal pupal development in *HDAC3* knockdown insects. In general, histone acetylation and deacetylation at the promoter region are associated with transcription activation and repression, respectively [39]. Interestingly, differential gene expression analysis of sequences of RNA isolated from dsHDAC3 and dsmalE treated larvae identified 563 (76%) up-regulated, and 178 (24%) down-regulated genes. The data suggest that the HDAC3 is involved in suppression of gene expression in *T. castaneum* larvae. The maintenance of equilibrium between acetylation and deacetylation of histones and non-histone proteins is essential for healthy cell growth. Loss of HDAC1 or HDAC3 leads to cell growth inhibition and overexpression of genes involved in lipid metabolism, DNA replication, cell cycle regulation, and signal transduction [13]. Histone acetyltransferases and deacetylases control cell proliferation and differentiation [40].

Myo22, a myosin heavy chain gene essential for muscle development, is a common gene up-regulated by *HDAC3* knockdown larvae, and TSA treated cells. (Additional file 4, Fig. 5A, B). Functions of myosin heavy chain (MHC) family genes in *T. castaneum* were reported recently [41]. The TcMyo20 is essential for wing and leg morphogenesis in *T. castaneum*. MHC isoforms regulate muscle function and are critical for specialized functions such as flying and jumping in *D. melanogaster* [42]. The activity of HDACs (class I, II) promoted swimming performance, but reduced slow and fast MHC content in cardiac and skeletal muscles in zebrafish [43] Trichostatin A (TSA, Class I, II HDAC inhibitor) induces JH response genes in a dose-dependent manner in *T. castaneum* [27]. TSA works as an epigenetic modulator, and deacetylation by TSA regulates the expression of key players involved in JH and 20E action in TcA cells [31]. HDAC inhibition by TSA led to an increase in the concentration of MHC in both skeletal and cardiac muscle in zebrafish (*Danio rerio*) [43]. Histone acetyltransferases CBP/p300 binds to the muscle-specific promoter, and this leads to enhanced transcription of muscle-specific genes [40]. Our data suggest that HDAC3 could play an important role in muscle development and function. However, further studies are needed to identify the exact mechanism of HDAC3 suppression of Myo22 in *T. castaneum*.

Our results also demonstrated the role of HDAC3 in the regulation of ‘shaven,’ a transcription factor that is involved in the development of a variety of sensory organs [44]. Additionally, we confirmed the upregulation of Pvf3 in *HDAC3* knockdown larvae. Pvf3 functions in embryonic plasmatocyte survival and migration in *D. melanogaster* [45]. Interestingly, LOC103313779 (nuclear receptor corepressor 1, TC006021; *Drosophila* orthologue for smrter (smr), corepressor of ecdysone receptor) was up-regulated in *HDAC3* knockdown samples (Additional file 1, Table S3). Suppression of *HDAC3* enhances apoptosis induced by paclitaxel in human maxillary cancer cells [46].

Histone deacetylase 3 is associated with the nuclear receptor corepressor complex containing N-CoR and SMRT (Silencing mediator for retinoid and thyroid hormone receptors) [47, 48]. HDAC3 is crucial for repression by multiple nuclear receptors, and the N-CoR-HDAC3 complex plays a unique role in thyroid hormone receptor-mediated gene repression in human 232 T cells [49]. Our previous studies demonstrated that the HDAC1/SIN3 multiprotein complex regulates the expression of *Kr-h1* [30]. Further studies with the NCoR-HDAC3 complex is required to identify the mechanism of gene regulation by HDAC3.

In *D. melanogaster,* knockdown of RPD3 by RNAi affects global histone acetylation, especially K9/14 of histone H3 and K8/K12 of histone H4 [50]. Similarly, HDAC1 and RPD3 disruptions result in histone H4 and H3 hyperacetylation, especially at H3K9/18 and H4K5 and K12 in the *Saccharomyces cerevisiae* [51]. Our western blot results showed that RNAi- mediated knockdown of *HDAC3* results in an increase in acetylation of Histone H3K9 and H3K27 in *T. castaneum* larvae (Fig. 6). In rats, acetylation of H3K9 increased in cortical neurons accompanied by a reduction of nuclear localization of HDAC3 [52]. Treatment of HeLa cells with TSA or silencing of *HDAC3* expression by small interfering RNA caused hyperacetylation of Lys-9 in histone H3 near the growth-differentiation factor 11 (gdf11) promoter [53]. HDAC3 selectively represses CREB3-mediated transcription and migration of metastatic breast cancer cells [54]. The histone H3 acetylase dGcn5 is a notable player in *D. melanogaster* metamorphosis [55]. HDAC3 deacetylases myocyte enhance factor 2, a transcription factor essential for controlling gene expression, muscle differentiation, apoptosis, and survival of different cell types [33]. These data suggest that *HDAC3* suppression induces the acetylation status of histone H3. WEGO plot showed enrichment of terms involved in biological regulation, regulation of the cellular process, signal transduction, ion binding, and catalytic activity in *HDAC3* knockdown insects.

## Conclusions

*HDAC3* knockdown interferes with the JH response gene *Kr-h1,* pupal specifier *Br-C,* and the adult specifier transcription factor *E93*. JH response gene Kr-h1 and Br-C was significantly up-regulated by *HDAC3* knockdown; the ecdysone response gene E93 was significantly down-regulated. Six genes were commonly up-regulated in dsHDAC3- and TSA-treatment, and down-regulated in dsCBP treatment (Additional file 1, Table S8). We further confirmed our result with RT-qPCR and identified that TC003570 (Shaven) and TC008417 (pvf3), which are important for sensory and cell proliferation, respectively, are significantly up-regulated in *HDAC3* knockdown pupae. A model for the HDAC3 function is shown in Additional file 1, Fig. S6. In conclusion, we identified that HDAC3 reduction in *T. castaneum* affects the genes responsible for muscle development and signal transduction, and thereby affecting the development and metamorphosis.

## Methods

### Insect rearing and cell culture

*T. castaneum* GA-1 strain [56] beetles were reared on organic wheat flour (Heartland Mill, Marienthal, KS) containing 10% dried baker yeast (MP biomedicals, Solon, OH) at 30 °C and 65 ± 5% relative humidity. The *T. castaneum* cells (BCIRL-TcA-CLG1, TcA) were grown in EX-CELL 420 (Sigma-Aldrich, St-Louis, MO) medium supplemented with 10% Fetal Bovine Serum (FBS, VWR-Seradigm, Radnor, PA) at 28 °C [57].

### Hormone treatments

Insect growth regulator/JH analog, S-Hydroprene (Sigma-Aldrich, MO), was used to test the response of the *HDAC3* gene to JH. Cyclohexane was used as a solvent control where hydroprene was dissolved to obtain the concentration of 2 μg/μl, and 1 μg (0.5 μl/larvae) hydroprene was topically applied to 48 h-old final instar larvae for in vivo hormone treatments. Six hours after treatment, the samples were collected and analyzed by RT-qPCR. The relative HDAC3 and Kr-h1 mRNA were determined.

### Double-stranded RNA synthesis (dsRNA) treatment and differential gene expression analysis

RNA isolation, cDNA synthesis, quantitative reverse transcription PCR (RT-qPCR), Double-stranded RNA synthesis (dsRNA) and microinjection, RNA-sequencing (RNA-seq) and data analysis and annotations were performed as described our previous publications [26, 30, 31]. RT-qPCR was performed using gene-specific primers (Additional file 1, Table S1) and iTaq Universal SYBR Green Supermix (Bio-Rad, Hercules, CA) in Applied Biosystems StepOnePlus Real-time PCR instrument. The libraries for RNA sequencing were prepared, as described previously [26]. The pooled libraries were sequenced using the Illumina Hiseq 4000 sequencer at Duke University Sequencing and Genomic Technologies (NC, USA). The raw reads were demultiplexed, trimmed, and transcripts were mapped back to the *T. castaneum* reference genome (assembly Tcas5.2) using the CLC genomic workbench pipeline (Version 11.0.1).

### Protein extraction and western blotting

The larvae were cleaned with ice-cold PBS and lysed in RIPA lysis buffer (Radioimmunoprecipitation assay buffer- 0.5 M Tris-HCl, pH 7.4, 1.5 M NaCl, 2.5% deoxycholic acid, 10% NP-40, 10 mM EDTA- Millipore Sigma, MA, USA) containing Halt protease inhibitor cocktail (ThermoFisher, Rockford, IL, USA). The lysate was clarified in a microfuge at 8000 rpm for 5 min. The proteins in the supernatant were precipitated by adding in order 1:8:1 ratio of lysate: ice-cold acetone: Trichloroacetic acid (TCA), and incubating at − 20 °C overnight. The solution was then centrifuged in a microfuge at 12000 rpm for 20 min. The pellet was washed in ice-cold acetone twice to remove the TCA traces, air-dried, and dissolved in 10% SDS. The proteins quantified using Bio-Rad protein assay concentrate and standard. Samples were denatured (95–100 °C for 5 min) in 5X SDS loading buffer to denature and stored at − 20 °C for future use. An equal amount of proteins were loaded and resolved on 12% SDS-polyacrylamide gels, along with the protein ladder. The proteins were transferred from the gel to the polyvinylidene difluoride (PVDF) membranes (Bio-Rad, Hercules, CA) and blocked with 5% nonfat dried milk blocking buffer for one hour at room temperature. The membranes were washed and incubated with 1000x diluted primary lysine-acetylated antibody-Histone H3 antibody sampler kit #9927 (Cell Signaling Technology, Danvers, MA) overnight at 4 °C with gentle shaking. The membranes were washed three times in Tris-buffered saline with Tween 20 (TBST) and incubated with 1000x diluted HRP-conjugated secondary antibody (#7074- Cell Signaling) in blocking buffer at room temperature for one hour as described previously [58]. The signals were developed with Supersignal West Femto Maximum sensitivity Substrate (ThermoFisher, IL), following the manufacturer’s protocol. The images were acquired in a darkroom using the chemiluminescence technique. Western blot bands were quantified by Image-J software and normalized with ß-Actin loading control protein.

### Statistical analysis

JMP Pro 14.0 (SAS Institute Inc., Cary, NC) software was used for *t*-test, comparing means, *P*-value < 0.05.

## Supplementary information

**Additional file 1.**

**Additional file 2.**

**Additional file 3.**

**Additional file 4.**

**Additional file 5.**

## Data Availability

Short-read (Illumina HiSeq 4000) sequence data are available in the NCBI SRA (accession numbers PRJNA634129). BioSample metadata are available in the NCBI BioSample database (http://www.ncbi.nlm.nih.gov/biosample/) under accession number SAMN14984385, SAMN14984386, SAMN14984388, SAMN14984390).

## Abbreviations

HDAC3: Histone deacetylase 3
JH: Juvenile hormone
SRC: Steroid receptor co-activator
CBP: CREB-binding protein
TSA: Trichostatin A
4EBP: Eukaryotic translation initiation factor 4E-binding protein 2
E93: Transcription factor E93
Kr-h1: Krüppel homolog 1
20E: 20-hydroxyecdysone
DEG: Differentially expressed gene
dsRNA: Double-stranded RNA

## References

[1] Kouzarides T (2007). Chromatin modifications and their function. Cell.

[2] Marmorstein R, Zhou MM (2014). Writers and readers of histone acetylation: structure, mechanism, and inhibition. Cold Spring Harb Perspect Biol.

[3] Seto E, Yoshida M (2014). Erasers of histone acetylation: the histone deacetylase enzymes. Cold Spring Harb Perspect Biol.

[4] Choudhary C, Kumar C, Gnad F, Nielsen ML, Rehman M, Walther TC, Olsen JV, Mann M (2009). Lysine acetylation targets protein complexes and co-regulates major cellular functions. Science.

[5] de Ruijter AJ, van Gennip AH, Caron HN, Kemp S, van Kuilenburg AB (2003). Histone deacetylases (HDACs): characterization of the classical HDAC family. Biochem J.

[6] Haberland M, Montgomery RL, Olson EN (2009). The many roles of histone deacetylases in development and physiology: implications for disease and therapy. Nat Rev Genet.

[7] Lagger G, O'Carroll D, Rembold M, Khier H, Tischler J, Weitzer G, Schuettengruber B, Hauser C, Brunmeir R, Jenuwein T (2002). Essential function of histone deacetylase 1 in proliferation control and CDK inhibitor repression. EMBO J.

[8] Marks PA (2010). Histone deacetylase inhibitors: a chemical genetics approach to understanding cellular functions. Biochim Biophys Acta.

[9] Delcuve GP, Khan DH, Davie JR (2012). Roles of histone deacetylases in epigenetic regulation: emerging paradigms from studies with inhibitors. Clin Epigenetics.

[10] Bhaskara S, Chyla BJ, Amann JM, Knutson SK, Cortez D, Sun ZW, Hiebert SW (2008). Deletion of histone deacetylase 3 reveals critical roles in S phase progression and DNA damage control. Mol Cell.

[11] Cho Y, Griswold A, Campbell C, Min KT (2005). Individual histone deacetylases in *Drosophila* modulate transcription of distinct genes. Genomics.

[12] Johnson CA, Barlow AL, Turner BM (1998). Molecular cloning of Drosophila melanogaster cDNAs that encode a novel histone deacetylase dHDAC3. Gene.

[13] Foglietti C, Filocamo G, Cundari E, De Rinaldis E, Lahm A, Cortese R, Steinkuhler C (2006). Dissecting the biological functions of *Drosophila* histone deacetylases by RNA interference and transcriptional profiling. J Biol Chem.

[14] Gryder BE, Wu L, Woldemichael GM, Pomella S, Quinn TR, Park PMC, Cleveland A, Stanton BZ, Song Y, Rota R (2019). Chemical genomics reveals histone deacetylases are required for core regulatory transcription. Nat Commun.

[15] Zhu CC, Bornemann DJ, Zhitomirsky D, Miller EL, O'Connor MB, Simon JA (2008). *Drosophila* histone deacetylase-3 controls imaginal disc size through suppression of apoptosis. PLoS Genet.

[16] Bodai L, Zsindely N, Gaspar R, Kristo I, Komonyi O, Boros IM (2012). Ecdysone induced gene expression is associated with acetylation of histone H3 lysine 23 in *Drosophila melanogaster*. PLoS One.

[17] Jindra M, Palli SR, Riddiford LM (2013). The juvenile hormone signaling pathway in insect development. Annu Entomol.

[18] D.L. Denlinger GDY, J.P. Rinehart: Hormonal control of diapause. Insect Endocrinology, Academic Press*,* 2012:430–463.

[19] Goodman WG, and Cusson, M: The juvenile hormones. *Insect Endocrinology* 2012( Web.):310–365.

[20] Chippendale GM, Yin CM (1975). Reappraisal of proctodone involvement in the hormonal regulation of larval diapause. Biol Bull.

[21] Riddiford LM (2012). How does juvenile hormone control insect metamorphosis and reproduction?. Gen Comp Endocr.

[22] Ashok M, Turner C, Wilson TG (1998). Insect juvenile hormone resistance gene homology with the bHLH-PAS family of transcriptional regulators. Proc Natl Acad Sci U S A.

[23] Charles JP, Iwema T, Epa VC, Takaki K, Rynes J, Jindra M (2011). Ligand-binding properties of a juvenile hormone receptor, Methoprene-tolerant. Proc Natl Acad Sci U S A.

[24] Zhang ZL, Xu JJ, Sheng ZT, Sui YP, Palli SR (2011). Steroid receptor co-activator is required for juvenile hormone signal transduction through a bHLH-PAS transcription factor, Methoprene tolerant. J Biol Chem.

[25] Fernandez-Nicolas A, Belles X (2016). CREB-binding protein contributes to the regulation of endocrine and developmental pathways in insect hemimetabolan pre-metamorphosis. Biochim Biophys Acta.

[26] Roy A, George S, Palli SR (2017). Multiple functions of CREB-binding protein during postembryonic development: identification of target genes. BMC Genomics.

[27] Xu J, Roy A, Palli SR (2018). CREB-binding protein plays key roles in juvenile hormone action in the red flour beetle, *Tribolium castaneum*. Sci Rep.

[28] Minakuchi C, Namiki T, Shinoda T (2009). Kruppel homolog 1, an early juvenile hormone-response gene downstream of Methoprene-tolerant, mediates its anti-metamorphic action in the red flour beetle *Tribolium castaneum*. Dev Biol.

[29] Jindra M, Belles X, Shinoda T (2015). Molecular basis of juvenile hormone signaling. Curr Opin Insect Sci.

[30] George S, Gaddelapati SC, Palli SR (2019). Histone deacetylase 1 suppresses Krüppel homolog 1 gene expression and influences juvenile hormone action in *Tribolium castaneum*. Proc Natl Acad Sci U S A.

[31] Roy A, Palli SR (2018). Epigenetic modifications acetylation and deacetylation play important roles in juvenile hormone action. BMC Genomics.

[32] Vanhaecke T, Papeleu P, Elaut G, Rogiers V (2004). Trichostatin A-like hydroxamate histone deacetylase inhibitors as therapeutic agents: toxicological point of view. Curr Med Chem.

[33] Grégoire S, Xiao L, Nie J, Zhang X, Xu M, Li J, Wong J, Seto E, Yang XJ (2007). Histone deacetylase 3 interacts with and deacetylates myocyte enhancer factor 2. Mol Cell Biol.

[34] Chuang HC, Chang CW, Chang GD, Yao TP, Chen H (2006). Histone deacetylase 3 binds to and regulates the GCMa transcription factor. Nucleic Acids Res.

[35] Ozawa T, Mizuhara T, Arata M, Shimada M, Niimi T, Okada K, Okada Y, Ohta K (2016). Histone deacetylases control module-specific phenotypic plasticity in beetle weapons. Proc Natl Acad Sci U S A.

[36] Chen M, Zhang N, Jiang H, Meng X, Qian K, Wang J (2019). Molecular characterization of class I histone deacetylases and their expression in response to thermal and oxidative stresses in the red flour beetle, *Tribolium castaneum*. Genetica.

[37] Urena E, Manjon C, Franch-Marro X, Martin D (2014). Transcription factor E93 specifies adult metamorphosis in hemimetabolous and holometabolous insects. Proc Natl Acad Sci U S A.

[38] Urena E, Chafino S, Manjon C, Franch-Marro X, Martin D (2016). The occurrence of the Holometabolous Pupal stage requires the interaction between E93, Kruppel-homolog 1 and broad-complex. PLoS Genet.

[39] Swaminathan A, Gajan A, Pile LA: Epigenetic regulation of transcription in *Drosophila*. Front Biosci (Landmark Ed) 2012, 17:909–937. 10.2741/3964 22201781

[40] Lehrmann H, Pritchard LL, Harel-Bellan A (2002). Histone acetyltransferases and deacetylases in the control of cell proliferation and differentiation. Adv Cancer Res.

[41] Li C, Liu J, Lu P, Ma S, Zhu K, Gao L, Li B, Chen K (2019). Identification, expression and function of myosin heavy chain family genes in *Tribolium castaneum*. Genomics.

[42] Wells L, Edwards KA, Bernstein SI (1996). Myosin heavy chain isoforms regulate muscle function but not myofibril assembly. EMBO J.

[43] Seebacher F, Simmonds AIM (2019). Histone deacetylase activity mediates thermal plasticity in zebrafish (*Danio rerio*). Sci Rep.

[44] Kavaler J, Fu W, Duan H, Noll M, Posakony JW (1999). An essential role for the *Drosophila* Pax2 homolog in the differentiation of adult sensory organs. Development.

[45] Duchek P, Somogyi K, Jekely G, Beccari S, Rorth P (2001). Guidance of cell migration by the *Drosophila* PDGF/VEGF receptor. Cell.

[46] Narita N, Fujieda S, Kimura Y, Ito Y, Imoto Y, Ogi K, Takahashi N, Tanaka T, Tsuzuki H, Yamada T (2010). Suppression of histone deacetylase 3 (HDAC3) enhances apoptosis induced by paclitaxel in human maxillary cancer cells in vitro and in vivo. Biochem Biophys Res Commun.

[47] Glass CK, Rosenfeld MG (2000). The coregulator exchange in transcriptional functions of nuclear receptors. Genes Dev.

[48] Kao HY, Downes M, Ordentlich P, Evans RM (2000). Isolation of a novel histone deacetylase reveals that class I and class II deacetylases promote SMRT-mediated repression. Genes Dev.

[49] Ishizuka T, Lazar MA (2003). The N-CoR/histone deacetylase 3 complex is required for repression by thyroid hormone receptor. Mol Cell Biol.

[50] Pile LA, Schlag EM, Wassarman DA (2002). The SIN3/RPD3 deacetylase complex is essential for G (2) phase cell cycle progression and regulation of SMRTER corepressor levels. Mol Cell Biol.

[51] Rundlett SE, Carmen AA, Kobayashi R, Bavykin S, Turner BM, Grunstein M (1996). HDA1 and RPD3 are members of distinct yeast histone deacetylase complexes that regulate silencing and transcription. Proc Natl Acad Sci U S A.

[52] Yang X, Wu Q, Zhang L, Feng L (2016). Inhibition of histone deacetylase 3 (HDAC3) mediates ischemic preconditioning and protects cortical neurons against ischemia in rats. Front Mol Neurosci.

[53] Zhang X, Wharton W, Yuan Z, Tsai SC, Olashaw N, Seto E (2004). Activation of the growth-differentiation factor 11 gene by the histone deacetylase (HDAC) inhibitor trichostatin a and repression by HDAC3. Mol Cell Biol.

[54] Kim HC, Choi KC, Choi HK, Kang HB, Kim MJ, Lee YH, Lee OH, Lee J, Kim YJ, Jun W (2010). HDAC3 selectively represses CREB3-mediated transcription and migration of metastatic breast cancer cells. Cell Mol Life Sci.

[55] Carre C, Szymczak D, Pidoux J, Antoniewski C (2005). The histone H3 acetylase dGcn5 is a key player in *Drosophila melanogaster* metamorphosis. Mol Cell Biol.

[56] Haliscak JP, Beeman RW (1983). Status of malathion resistance in five genera of beetles infesting farm-stored corn, wheat, and oats in the United States. J Econ Entomol.

[57] Goodman CL, Stanley D, Ringbauer JA, Beeman RW, Silver K, Park Y (2012). A cell line derived from the red flour beetle *Tribolium castaneum* (Coleoptera: Tenebrionidae). In Vitro Cell Dev Biol Anim.

[58] Gaddelapati SC, Dhandapani RK, Palli SR (1863). CREB-binding protein regulates metamorphosis and compound eye development in the yellow fever mosquito, Aedes aegypti. Biochimica et Biophysica Acta (BBA) - Gene Regulatory Mechanisms.

