# Supplementary material for: Histone deacetylase 3 is required for development and metamorphosis in the red flour beetle, Tribolium castaneum
Source: BMC Genomics. 2020 Jun 22;21:420. doi: 10.1186/s12864-020-06840-3 (PMC7310253; doi:10.1186/s12864-020-06840-3)
Supplement: Supplementary file 1 — Additional file 1. [file 12864_2020_6840_MOESM1_ESM.pdf]

# Histone deacetylase 3 is a crucial regulator for metamorphosis and muscle development in the red flour beetle, *Tribolium castaneum* (Herbst)

Smitha George and Subba Reddy Palli*

Department of Entomology, University of Kentucky, Lexington, Kentucky 40546, USA

*Corresponding author

Phone: 859-257-4962

Fax: 859-323-1120

Email: rpalli@email.uky.edu

List of materials included

Figure S1. Phylogenetic tree of HDACs families in the *T. castaneum.*

Figure S2. HDAC3 pupal phenotypes.

Figure S3. Tissue-specific expression of HDAC3 in *T. castaneum* larvae and pupae

Figure S4. Volcano Plot and WEGO Plot

Figure S5. Venn diagrams comparing the upregulated genes from dsHDAC3 (larvae) and Trichostatin A (TSA) treated TcA cells.

Figure S6. Schematic representation of HDAC3 action in the red flour beetle.

Table S1. List of dsRNA and RT-qPCR primers used in this study.

Table S2. Summary statistics of RNA-sequencing data.

Table S3. Expression changes of hormone-related genes identified by RNA-seq analysis.

Table S4. The list of select differentially expressed genes identified by RNA-seq and verified by RT-qPCR in *HDAC3* knockdown larvae.

Table S5. Upregulated JH-response genes in *HDAC3* knockdown larvae.

Table S6. Zn-Finger (COG5048) domains identified among upregulated genes in dsHDAC3 treated larvae.

Table S7. List of genes selected from Additional file 3 (comparing TSA induced genes (TcA cells) with upregulated genes in *HDAC3* knockdown larvae) for RT-qPCR verification.

Table S8. Common genes identified among those upregulated after HDAC3 knockdown induced by TSA and down-regulated after CBP knockdown.

Additional file 2 Excel file: Details of 741 DEG identified after HDAC3 knockdown ( P-value <0.05, 2 fold)

Additional file 3 Excel file: Details of 148 DEG identified after HDAC3 knockdown (P-value <0.01, 4 fold)

Additional file 4 Excel file: A) Details of common upregulated genes identified from HDAC3 knockdown larvae and TSA treatments (TcA cells). B) Details of common genes identified among dsHDAC3 (upregulated) vs. dsCBP (downregulated) vs. TSA (upregulated)

Additional file 5: Complete images of Western blots shown in Figure 6

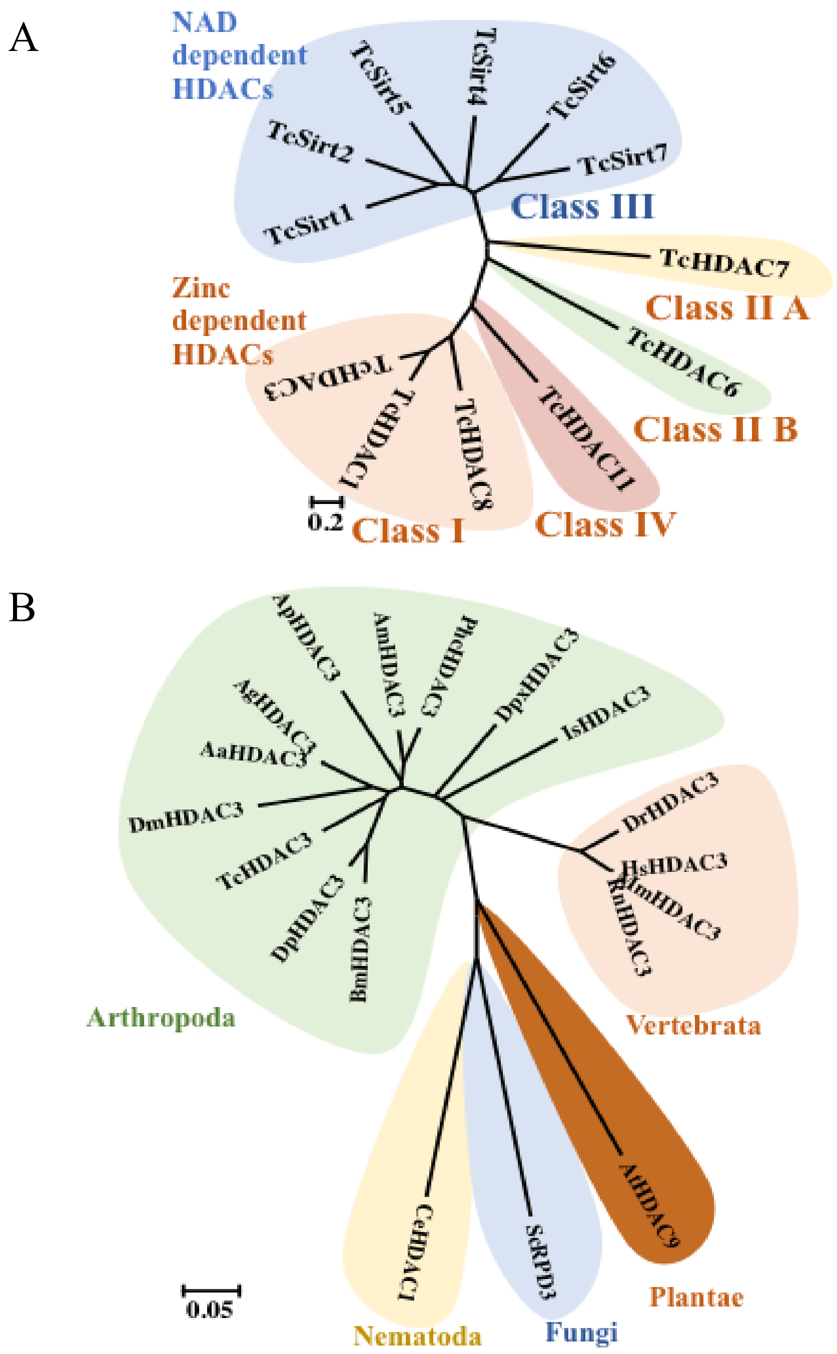


**Fig. S1. HDAC3 is conserved and universal.**

A) **Phylogenetic tree of HDACs families in the *T. castaneum*.** The evolutionary HDAC tree was inferred using the Neighbor-joining method. Evolutionary analyses were conducted in MEGA7 [1]. The tree is drawn to scale, with branch lengths in the same units as those of the evolutionary distances used to infer the phylogenetic tree. The evolutionary distances were computed using the

Poisson correction method and the units of the number of amino acid substitutions per site. B) Neighbor-joining phylogenetic tree of HDAC3 constructed using the amino acid sequence of proteins from selected organisms. (**Arthropoda:** *Drosophila melanogaster* (Dm) (Insecta: Diptera), *Ixodes scapularis* (Arachnida: Ixodida), *Pediculus humanus capitis* (Insecta: Phthiraptera), *Apis mellifera* (Insecta: Hymenoptera), *Tribolium castaneum* (Insecta: Coleoptera), *Acyrthosiphon pisum* (Insecta: Homoptera), *Bombyx mori* (Insecta: Lepidoptera), *Aedes aegypti* (Insecta: Diptera), *Anopheles gambiae* (Insecta: Diptera), *Daphnia pulex* (Crustacea: Water flea). **Vertebrata:** *Homo sapiens* (Hs), *Danio rerio* (Dr), *Rattus norvegicus* (Rn), *Mus musculus* (Mm). **Nematoda**: *Caenorhabditis elegans* (Ce). **Fungi:** *Saccharomyces cerevisiae* (Sc) (Yeast). **Plantae:** *Arabidopsis thaliana* (At).

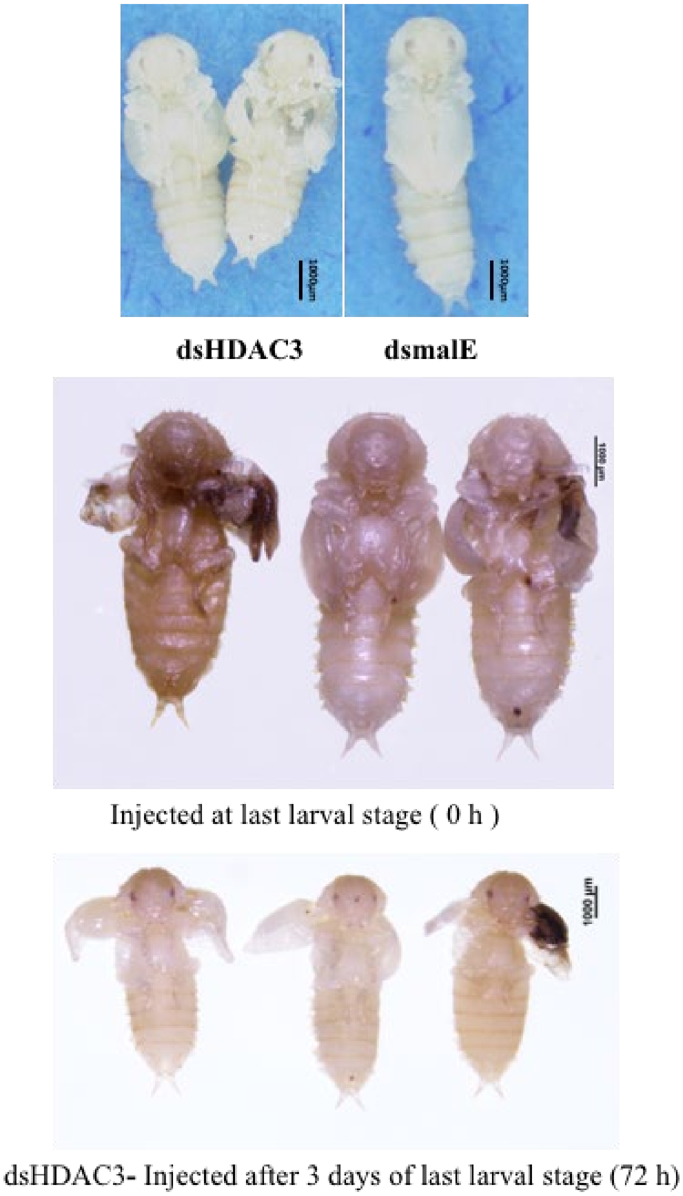


**Fig. S2. HDAC3 knockdown induced pupal phenotypes.**

Phenotypes observed in insects injected with dsHDAC3 into the last instar larvae. Developmental defects of wings with sclerotized areas were detected.

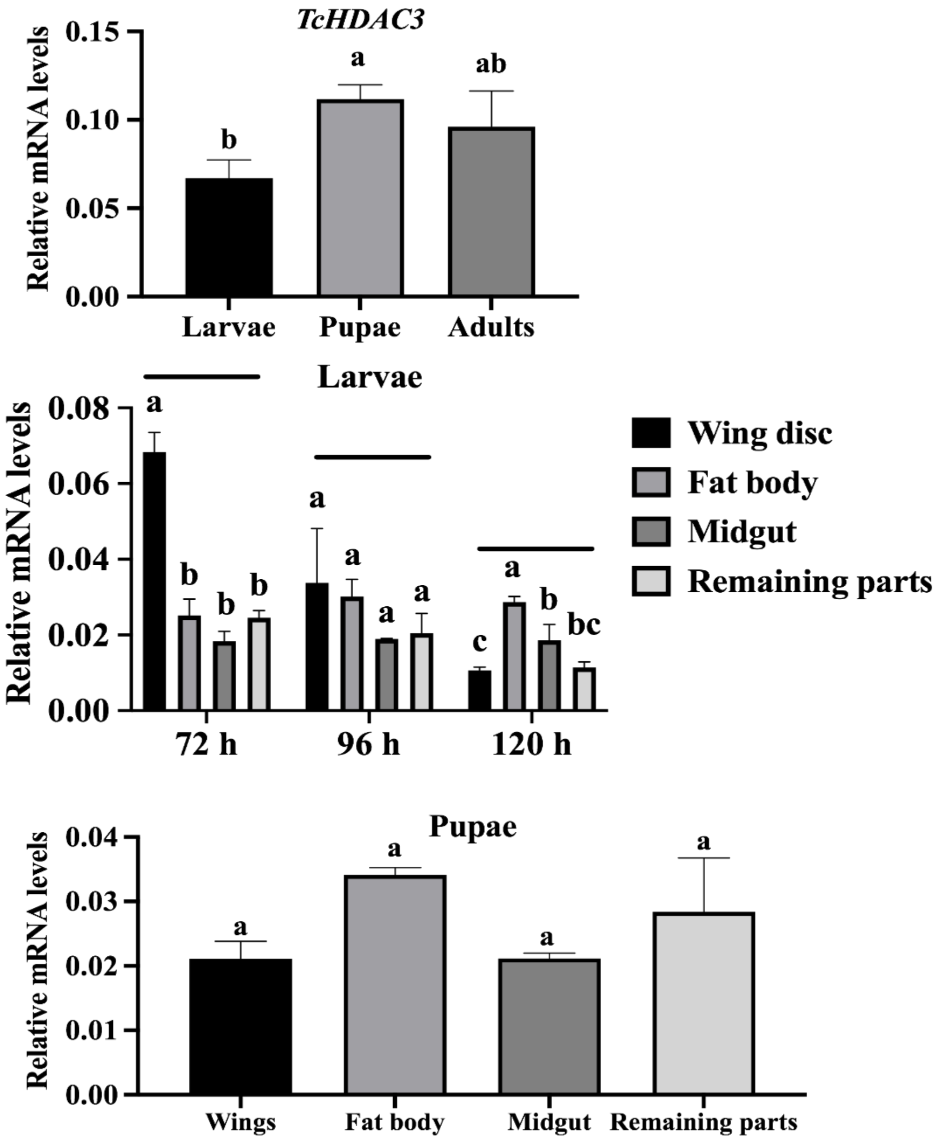


**Fig. S3. Tissue-specific expression of HDAC3 in *T. castaneum* larvae and pupae. Samples were collected from 72 h, 96 h and 120 h (day 3- day 5) old last instar larvae, pupae and adults. RNA was extracted and converted cDNA and used to determine relative mRNA levels of HDAC3.**

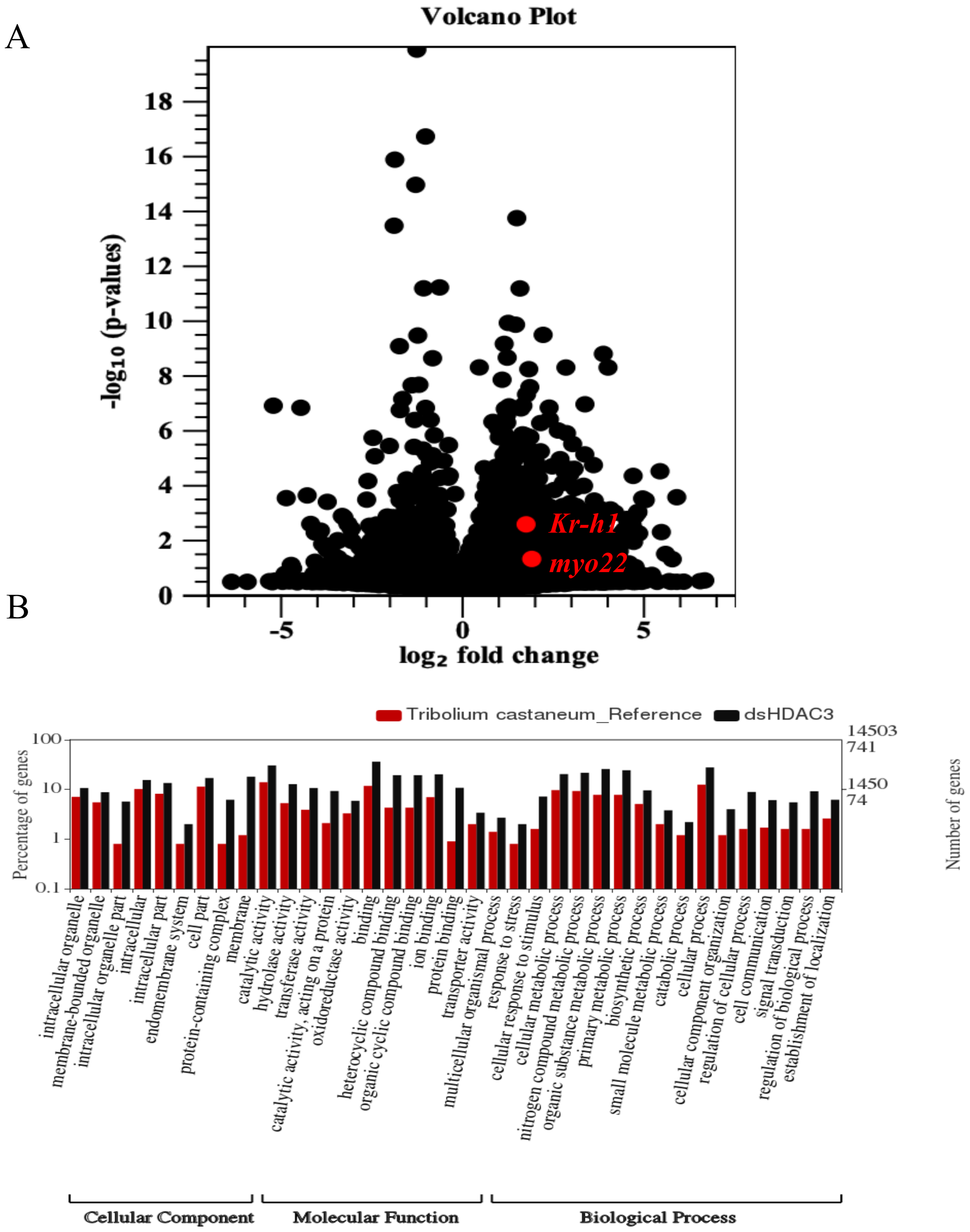


**Figure S4. Volcano Plot and WEGO Plot.** A) Volcano plot highlighting Kr-h1 and myo22. B) The WEGO analysis identified enrichment of various biological processes, molecular and cellular functions. The WEGO histogram shows the number and percent of genes in the cellular

component, molecular component, and biological function ontology classification group for *T. castaneum* transcriptome and 741 upregulated DEG genes in *HDAC3* knockdown larvae. The Blast2GO PRO-plugin software was used for the functional annotation of the transcripts.

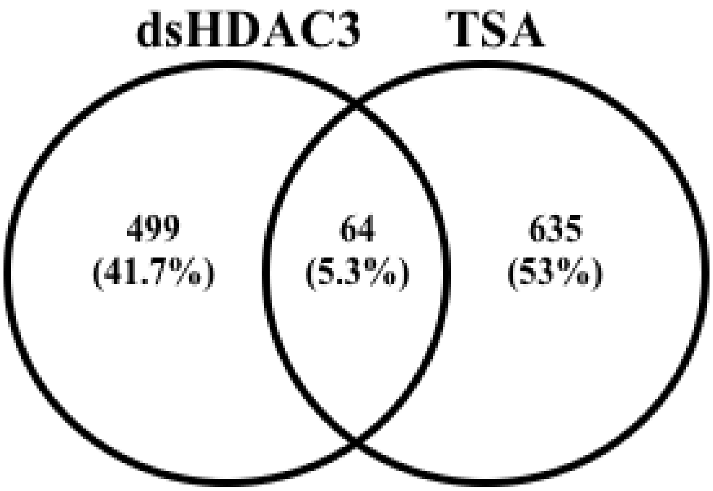


**Fig. S5. Venn diagrams comparing the upregulated genes from dsHDAC3 (larvae) and Trichostatin A (TSA) treated TcA cells.**

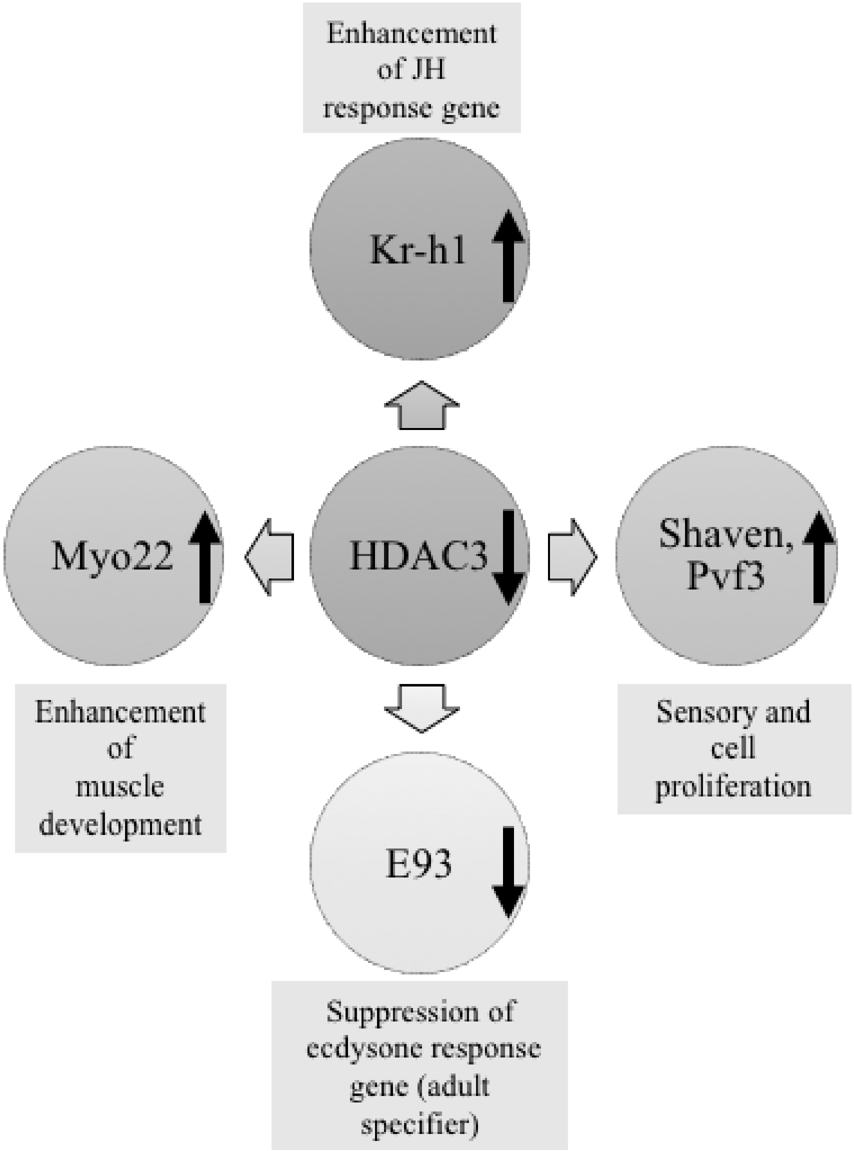


**Fig. S6. Schematic representation of HDAC3 action in the red flour beetle.**

JH response gene *Kr-h1* was significantly upregulated by HDAC3 knockdown; the ecdysone response gene E93 was significantly down-regulated. Myo22, a myosin heavy chain gene essential for muscle development, is upregulated by HDAC3 knockdown. TC003570 (Shaven) and TC008417 (pvf3), which are essential for sensory and cell proliferation, significantly upregulated in pupae developed from HDAC3 knockdown larvae.

**Additional Tables**

**Table S1: List of dsRNA and RT-qPCR primers used in this study.**

| Gene | Primer |
|---|---|
| TcdsHDAC3F | TAATACGACTCACTATAGGGTGCAGATTTACCGGCCTTAC |
| TcdsHDAC3R | TAATACGACTCACTATAGGGATGACCCGATCAGTGAGATAGA |
| TcqHDAC3F | GAGACGCAATCCCTGAAG |
| TcqHDAC3R | TTTCGCCTCGACCATCTTA |
| TcqMyo22F | TTGCTCTCTCTCGCATGTTC |
| TcqMyo22R | GCCTTCTTCGGTCACGTAAT |
| TcqShavenF | GGAGTGTTCGTTAACGGTAGG |
| TcqShavenR | CCCTCAGCTGCCTTGAAATA |
| TcqPvf3F | CCAGCTCCTGTCTCTTCTTTC |
| TcqPvf3R | CTCCAATGCGGCGTTTATTG |
| Tcq013029F | CTGATGAGCTTCTGGACGATAG |
| Tcq013029R | CTCGTCCAGTTCCAGCTAAAT |
| Tcq005670F | GAAGCCCTGCGACCAAATA |
| Tcq005670R | GTGTCGTCGTCGTTCTCTTTAC |
| Tcq012510F | CTCTCTGAAGCACCACAATGA |
| Tcq012510R | CGTTTCGACGTAGCCTTGAT |
| Tcq032605F | ACCGTACCAGTGTGAGAATTG |
| Tcq032605R | CGGAATAAGACCACACGTATCC |
| Tcq003005F | ATGCCTTCATCCAGCGTAAG |
| Tcq003005R | TTCACCGCTTGTAGAGGTTTC |
| Tcq000401F | AACGCCGACGTCACTTATTC |
| Tcq000401R | GCGCTCGTAGTCCAACATATC |

**Table S2: Summary statistics of RNA-sequencing data**

**A. Run Summary**

| Lane | PF* Yield (bp) | Number of PF* Clusters** | Q30% | Average Quality Score |
|---|---|---|---|---|
| 7 | 15,963,128,520 | 313,002,520 | 95.64 | 39.04 |

* PF: Passed Filter. ** For single-read sequencing (SR), number of reads = number of clusters.

**B. Read count statistics**

| Samples | Read Count | Single, mapped % | Reverse % of reads mapped |
|---|---|---|---|
| MalE-1 | 12,545,282 | 51.11 | 80.56 |
| MalE-2 | 16,562,495 | 45.23 | 82.45 |
| MalE-3 | 23,183,581 | 53.19 | 82.30 |
| HDAC3-1 | 14,874,511 | 65.17 | 84.52 |
| HDAC3-2 | 19,121,057 | 40.97 | 83.11 |
| HDAC3-3 | 8,396,093 | 49.12 | 83.07 |

**Table S3: Expression changes of hormone-related genes identified by RNA-seq analysis**

| Gene symbol | Gene description | fold change[a] | *P*-value[b] |
|---|---|---|---|
| *Kr-h1* | Krüppel homolog 1 | 3.36 | 0.00 |
| LOC655046 | Ecdysone induced protein 78C | 16.06 | 5E-09 |
| LOC660434 | Broad-complex core protein isoform 6-like | 2.40 | 0.03 |
| LOC103313779 | Nuclear receptor corepressor 1 | 2.69 | 0.00 |
| LOC658947 | Nuclear hormone receptor FTZ-F1 beta | 2.04 | 0.00 |

[a]Experiment - fold change

[b]Baggerley's test: normalized values

**Table S4. The list of select differentially expressed genes identified by RNA-seq and verified by RT-qPCR in *HDAC3* knockdown larvae.**

| Aliases | Fold change | Description | Domain |
|---|---|---|---|
| TC000110 | 5.2439 | transcription termination factor 2 | HELICc, DEXDc, SNF2_N |
| TC001775 | 2.8193 | nuclear factor 1 C-type | MH1 superfamily, NfI_DNAbd_pre-N |
| TC001921 | 6.4407 | lethal (3) malignant brain tumor-like protein 4 | MBT, SAM_Scm-like-3MBT3,4, zf-C2HC, SAM |
| TC003720 | 2.3232 | pangolin | SOX-TCF_HMG-box, CTNNB1_binding superfamily |
| TC003972 | 3.9383 | apterous a | Homeobox, LIM2_Lhx2_Lhx9, COG5576 |
| TC004015 | 8.8793 | uncharacterized LOC655848 | MBF2 |
| TC006021 | 2.6888 | nuclear receptor corepressor 1 | SANT superfamily |
| TC006040 | 2.3370 | homeobox protein PKNOX2 | Homeobox_KN, Meis_PKNOX_N |
| TC008129 | 2.3262 | N-alpha-acetyltransferase 30 | Acetyltransf_1, RimI |
| TC011468 | 2.9368 | mortality factor 4-like protein 1 | MRG, Tudor-knot |
| TC012866 | 4.6624 | FACT complex subunit Ssrp1 | HMG_box, PH2_SSRP1-like |
| TC014548 | 6.2153 | uncharacterized LOC100141687 | Zf-RING_2 |
| TC014708 | 3.7195 | uncharacterized LOC663601 (rel) | RHD_dimer, RHD-n super family |
| TC015048 | 3.0371 | zinc finger protein 184 | COG5048, zf-H2C2_2 |
| TC015577 | 2.1008 | pleiohomeotic | zf-C2H2, COG5048 |
| TC015843 | 3.1657 | uncharacterized LOC659528 | JHBP |
| TC016270 | 2.9061 | kelch-like ECH-associated protein 1 | BACK, BTB, Kelch, PHA03098 |
| TC031140 | 3.4616 | insulin-like growth factor 2 mRNA-binding protein 1 | KH-I |
| TC034026 | 2.3965 | broad-complex core protein isoform 6-like | BTB |
| TC012990 | 3.3629 | Krüppel homolog 1 | Zf-H2C2_2, COG5048 |

**Table S5: Upregulated JH-response genes in *HDAC3* knockdown larvae**

| Gene symbol | Gene description | JH III | | dsHDAC3 | |
|---|---|---|---|---|---|
| | | fold change | *P*-value | fold change | *P*-value |
| LOC659434 | fibroblast growth factor receptor homolog 1 | 96 | 0.030 | 4.1 | 0.004 |
| LOC100141923 | sodium-coupled monocarboxylate transporter 2-like | 203 | 0.004 | 3.8 | 0.009 |
| LOC103314154 | DNA primase large subunit | 3.8 | 0.020 | 3.4 | 0.011 |
| *Kr-h1* | Krüppel homolog 1 | 29.9 | 0.000 | 3.4 | 0.003 |
| LOC659929 | lachesin | 121 | 0.010 | 3.1 | 0.049 |
| LOC660154 | projectin | 14 | 0.030 | 3.0 | 0.000 |

[a]Experiment - Fold Change (normalized values)

[b]Baggerley's test: HDAC3 vs malE normalized values

**Table S6: Zn-Finger (COG5048) domains identified among upregulated genes in dsHDAC3 treated larvae**

| Gene Symbol | Locus tag | Gene description | fold change | *P*-value |
|---|---|---|---|---|
| *Kr-h1* | TC012990 | Krüppel homolog 1 | 3.36 | 0.00 |
| *Pho* | TC015577 | Pleiohomeotic | 2.10 | 0.01 |
| LOC662411 | TC015048 | zinc finger protein 184 | 3.04 | 0.03 |
| LOC662791 | TC002961 | transcriptional repressor CTCF | 2.26 | 0.00 |
| LOC103313118 | | gastrula zinc finger protein XlCGF57.1 | 2.89 | 0.01 |
| LOC103313190 | TC032605 | zinc finger protein 2-like | 13.87 | 0.00 |
| LOC103313193 | TC002118 | Krüppel like factor 6 | 2.30 | 0.01 |

**Table S7. List of genes selected from Appendix C** (comparing TSA induced genes (TcA cells) with upregulated genes in *HDAC3* knockdown larvae) **for RT-qPCR verification**

| | | **TSA-up** | | dsHDAC3-up | |
|---|---|---|---|---|---|
| **Locus tag** | **Gene Description** | **Fold change** | **P value** | **Fold change** | **P value** |
| TC012510 | uncharacterized LOC103314255 | 85.4897 | 0.0087 | 27.0809 | 0.0013 |
| TC032605 | zinc finger protein 2-like | 4.2700 | 0.0000 | 13.8733 | 0.0026 |
| TC003005 | muscle M-line assembly protein unc-89 | 3.1123 | 0.0000 | 10.3084 | 0.0000 |
| TC003570 | shaven | 2.5056 | 0.0001 | 7.7380 | 0.0223 |
| TC008923 | myo22 | 6.7952 | 0.0000 | 3.7507 | 0.0464 |
| TC013029 | neprilysin-11 | 2.5863 | 0.0000 | 2.0641 | 0.0356 |
| TC008417 | Pvf3 | 3.5201 | 0.0000 | 3.7258 | 0.0144 |
| TC000401 | FAT | 6.0517 | 0.0000 | 2.2999 | 0.0104 |
| TC005670 | epidermal growth factor-like protein 8 | 4.2720 | 0.0036 | 2.3405 | 0.0000 |

**Table S8. Common genes identified among those upregulated after HDAC3 knockdown, induced by TSA and down-regulated after CBP knockdown**

| | | TSA-up | | dsHDAC3-up | | dsCBP-down | |
|---|---|---|---|---|---|---|---|
| **Locus tag** | **Gene Description** | **Fold change** | ***P* value** | **Fold change** | ***P* value** | **Fold change** | ***P* value** |
| TC003570 | paired box protein Pax-5/sv/shaven | 2.51 | 0.00 | 7.74 | 0.02 | -2.07 | 0.00 |
| TC008417 | PDGF- and VEGF-related factor 3/Pvf3 | 3.52 | 0.00 | 3.73 | 0.01 | -2.47 | 0.00 |
| TC003987 | Titin | 5.46 | 0.00 | 2.75 | 0.00 | -2.20 | 0.00 |
| TC005670 | Epidermal growth factor-like protein 8 | 4.27 | 0.00 | 2.34 | 0.00 | -3.35 | 0.01 |
| TC000401 | FAT; Cadherin | 6.05 | 0.00 | 2.30 | 0.01 | -2.10 | 0.00 |
| TC013029 | Neprilysin-11 | 2.59 | 0.00 | 2.06 | 0.04 | -6.09 | 0.00 |

References


1. Kumar S, Stecher G, Tamura K: **MEGA7: Molecular evolutionary genetics analysis version 7.0 for bigger datasets**. *Mol Biol Evol* 2016, **33**(7):1870-1874.
