# Supplementary material for: Histone deacetylase 3 is required for development and metamorphosis in the red flour beetle, Tribolium castaneum
Source: BMC Genomics. 2020 Jun 22;21:420. doi: 10.1186/s12864-020-06840-3 (PMC7310253; doi:10.1186/s12864-020-06840-3)
Supplement: Supplementary file 5 — Additional file 5. [file 12864_2020_6840_MOESM5_ESM.pdf]

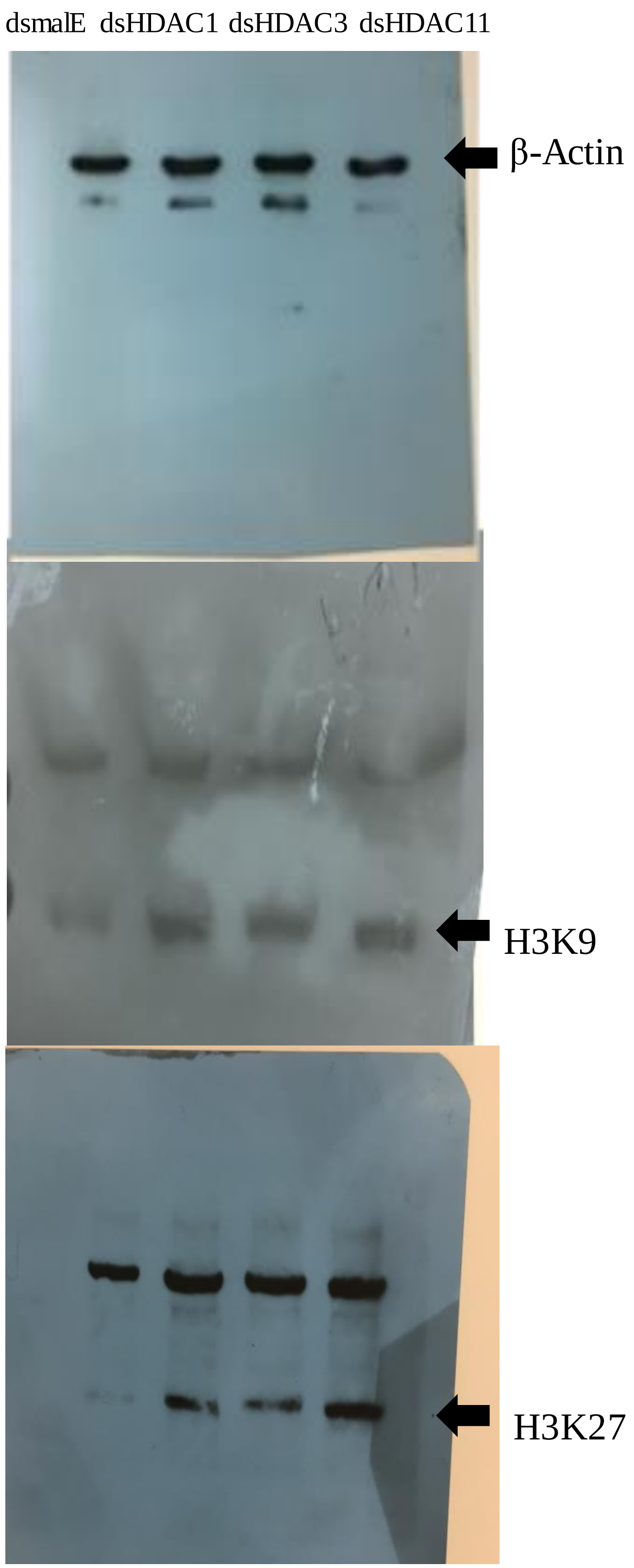
dsmalE dsHDAC1 dsHDAC3 dsHDAC11
β-Actin
H3K9
H3K27

Complete images of Western blots shown in Figure 6. The knockdown of HDAC1 and *HDAC3* increased acetylation levels of histone H3. Total protein extracted from dsHDAC1, dsHDAC3 or dsmalE injected larvae were separated on SDS-PAGE gels, transferred to western blots, and hybridized with antibodies recognizing Acetyl-Histone H3 (Antibody Sampler Kit # 9927-Cell Signaling. ß-actin served as a loading control. The HRP-linked IgG (#7074-Cell Signaling) was used as a secondary antibody.
